# Reviving the Dynamics of Attacked Reservoir Computers

**DOI:** 10.3390/e25030515

**Published:** 2023-03-16

**Authors:** Ruizhi Cao, Chun Guan, Zhongxue Gan, Siyang Leng

**Affiliations:** 1 Institute of AI and Robotics, Academy for Engineering and Technology, Fudan University, Shanghai 200433, China; 2 Research Institute of Intelligent Complex Systems, Fudan University, Shanghai 200433, China

**Keywords:** reservoir computer, attack and recovery, Echo State Property, network structure

## Abstract

Physically implemented neural networks are subject to external perturbations and internal variations. Existing works focus on the adversarial attacks but seldom consider attack on the network structure and the corresponding recovery method. Inspired by the biological neural compensation mechanism and the neuromodulation technique in clinical practice, we propose a novel framework of reviving attacked reservoir computers, consisting of several strategies direct at different types of attacks on structure by adjusting only a minor fraction of edges in the reservoir. Numerical experiments demonstrate the efficacy and broad applicability of the framework and reveal inspiring insights into the mechanisms. This work provides a vehicle to improve the robustness of reservoir computers and can be generalized to broader types of neural networks.

## 1. Introduction

Neural networks (NN) are subject to external perturbations and internal variations, especially when they are physically implemented [1,2,3,4]. In the past few decades, tremendous efforts have been devoted to relieving small perturbations on the input, that is, adversarial attacks [5,6,7], but are seldom made to consider the attack on the network structure. In fact, failure of certain neurons and/or synaptic connections may also significantly reduce the computational capacity [8,9], while biological NN compensate for this loss by adaptively adjusting/rebirthing links [10,11,12]. In clinical practice, neuromodulation techniques, such as the Transcranial Direct Current Stimulation (tDCS) [13,14,15], recover the neural functions through compensating neural connections with weak direct currents [16,17,18]. Restoring the network performance as much as possible from attacks on network structure becomes an urgent challenge. Albert et al. investigated the error tolerance and attack vulnerability under the removal of nodes in complex networks [19]. Nguyen et al. measured the network properties when a real-world complex network is attacked by different strategies [20]. Further studies concentrated on the recovery approaches [21,22], which are vital to the smooth functioning of the networked systems. However, the structural attack and recovery of neural networks are barely visited and require intensive investigation [23].

**Figure 1:**
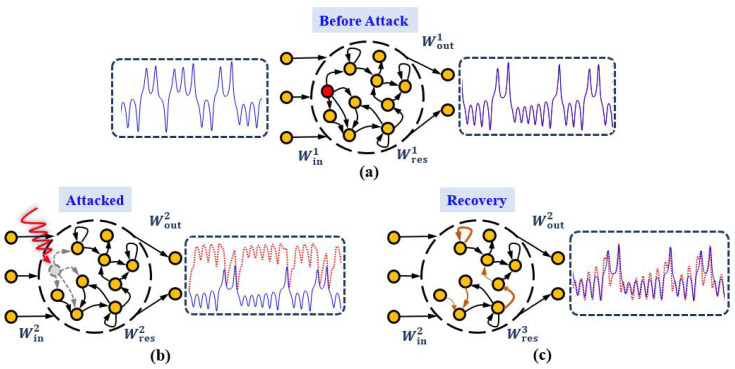
**Schematic diagram of attack and recovery in reservoir computer.** (**a**) Before attack, in the configuration of time series prediction, a standard RC accurately predicts the true dynamics, with blue and red lines denoting the true values and the predicted results respectively. (**b**) Attacked, here node attack is illustrated, failing its adjacent links (gray dashed arrows) and the predicted results deviate from the true values. (**c**) Recovery, by adjusting automatically part of the remaining links (orange arrows), the performance of RC improves significantly.

Reservoir Computer (RC), a variant of the Recurrent Neural Network (RNN), has enjoyed recent attention since the seminal works by Jaeger [24] and Maass [25], due to its excellent training efficiency and its convenience to be physically implemented [26]. Its architecture, comprising of an input layer, a linear output layer, and a reservoir network consisting of dynamical neurons, well-simulates the mechanism of biological brains in a conceptual manner [27]. While the input matrix and the reservoir matrix are randomly generated and fixed, training the output matrix occupies the whole expense, which can be efficiently obtained by least-squares optimization [28], and avails RC to reduce the complex training of RNN’s parameters to a linear regression problem [24]. The fixed weights also enable the reservoir layer to be created with a specific physical system [29]. Recent works analytically proved that a suitably trained RC is essentially a high-dimensional embedding of the input dynamical system [24,30,31,32,33,34], as shown in Figure 1a. When its reservoir becomes the target of an attack, on nodes and/or links, the performance may drop significantly and can not revive without external intervention [Figure 1b]. Similar operations performed in Deep Neural Networks (DNN) are known as *dropout* [35] and *pruning* [36], which are usually regarded as training tricks, instead of attacks, to improve the performance due to the redundant structures in DNN. Practically, the term “attacked” can be also interpreted/replaced as “failed” in broader scenarios. In physically implemented devices of RC, digital components, such as field-programmable gate arrays or digital signal processors, are used for the reservoir layer and readout layer. The memristor [29], a new type of information processing device which has a memory of past voltages or currents, is recently used to boost the power efficiency of the hardware implementations of reservoir computing systems. In these circumstances, the failures can usually be caused by the sudden disconnection between memristors during prolonged operations [37] or/and environmental damages to the internal electrical components [38]. These circumstances can be regarded as “attacks” to the reservoir and thus require “recovery”. Therefore, the study of attack and recovery for RC is not only at the theoretical level, but also has practical significance.

In this paper, analogous to the neural compensation mechanism in brains, we design several recovering mechanisms for reservoir computer to compensate its performance loss under different types of attacks on structure, that is, adjusting only a minor fraction of edges in the reservoir according to different attack scenarios. Results show that it is impossible to take precautions on specific nodes/links due to the ambiguous relationship between their *a priori* topological measurement and the performance loss under attack. Our proposed strategies successfully and efficiently revive the functioning of RC by automatically adjusting the remaining neurons/synapses [Figure 1c], which represent practical advancement towards enhancing the robustness of neural networks.

The paper is organized as follows: Section 2 reviews the standard reservoir computer and introduces the attack and recovery strategies employed in this study. Section 3 presents the performance loss under attack and the corresponding recovery results. We also quantitatively and systematically analyze the different recovery strategies. Section 4 discusses several important related issues and concludes the paper.

## 2. Method

### 2.1. Standard Reservoir Computer

The standard framework of RC can be described in the state updating rule of the reservoir neurons [24]:

(1) $$r_{k}=\left(1-\alpha \right)r_{k-1}+\alpha \phi \left(W_{\mathrm{res}}r_{k-1}+W_{\mathrm{in}}x_{k}\right),$$

where $r_{k}\in R^{m}$ represents the state of *m* reservoir neurons at time step *k*, and $x_{k}\in R^{n}$ is the input signal observed from a dynamical system φ evolving on a compact manifold M. $W_{\mathrm{in}}\in R^{m\times n}$ and $W_{\mathrm{res}}\in R^{m\times m}$ denote the input weight matrix and the reservoir network matrix respectively, which are randomly generated according to certain distribution laws and then fixed. We consider two settings, full connection and sparse connection, of the reservoir, while in the latter case two nodes i,j are called linked if $W_{\mathrm{res}}^{ij}\neq 0$ or $W_{\mathrm{res}}^{ji}\neq 0$ ($W_{\mathrm{res}}^{ij}$ denotes the link weight from node *i* to node *j*). α∈(0,1) is the leakage factor controlling the time-scale mismatch between the input and reservoir dynamics (α=1 represents the previous states do not leak into the current states) and function ϕ determines the dynamics of the reservoir neurons which at its simplest can be set as tanh(·). Consequently, the output $y_{k}\in R^{l}$ linearly combines the reservoir states such that $y_{k}=W_{\mathrm{out}}r_{k}$, with the output weight matrix $W_{\mathrm{out}}\in R^{l\times m}$ solely requiring training. RC can be adapted to different tasks while in the task of one-step time series prediction [39,40,41], the target is ${\hat{y}}_{k}:=x_{k+1}$ and $W_{\mathrm{out}}$ can be calculated by minimizing the loss function

(2) $$L=\sum_{k=1}^{N}{\left||x_{k+1}-W_{\mathrm{out}}r_{k}|\right|}^{2}+\beta {\left||W_{\mathrm{out}}|\right|}^{2},$$

where β>0 is the $L_{2}$-regularization coefficient. After the training phase, the output $y_{k}$ can be redirected to the input layer, that is, $x_{k+1}:=y_{k}$, thus RC runs in an autonomous mode (in this case l=n). With these settings, RC is proved to intactly capture the dynamics of the input dynamical system, which naturally requires the reservoir’s initial state to fade away, that is, the Echo State Property (ESP) [24,42]. A sufficient condition guaranteeing the ESP is that the spectral radius of $W_{\mathrm{res}}$ is smaller than 1, that is, $\left|\right|W_{\mathrm{res}}\left|\right|<1$. Thus in this study we rescale the reservoir network matrix by

(3) $$W_{\mathrm{res}}:=\frac{\rho^{*}}{\rho (W_{\mathrm{res}})}W_{\mathrm{res}},$$

where ρ(·) denotes the spectral radius or the maximum eigenvalue of a matrix, and $\rho^{*}$ is the desired spectral radius.

**Figure 2:**
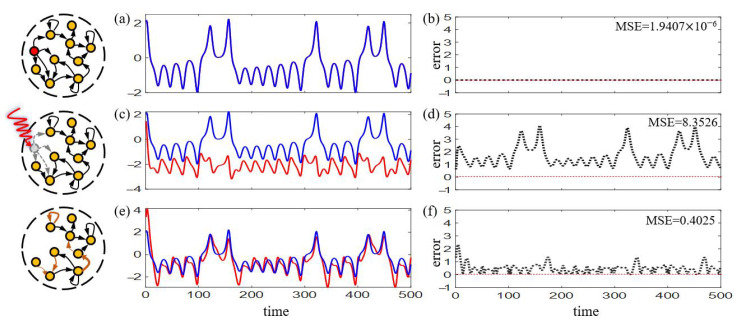
**Node-attack and recovery for fully connected RC.** (**a**) Before attack, predicted (**red**) and true (**blue**) time series. Here the two trajectories coincide with each other. (**c**) Attacked, predicted (**red**) and true (**blue**) time series. (**e**) Recovery, predicted (**red**) and true (**blue**) time series. (**b**,**d**,**f**) The prediction errors for each corresponding stage. Here the MSE value denotes an average of 50 realizations.

We use the Mean Squared Error (MSE) to evaluate RC’s performance at different stages:

(4) $${\mathrm{MSE}}_{k}:=\frac{1}{\tau }\sum_{p=k-\tau +1}^{k}\left|\right|y_{p}-{\hat{y}}_{p}\left|\right|,$$

where τ denotes a specified time window. Notice that a well-trained RC can achieve accurate prediction with saving computations [Figure 2a,b].

### 2.2. Reservoir-Attack Mechanisms

RC runs in an autonomous mode once the output matrix $W_{\mathrm{out}}$ is trained and fixed. We propose two possible attack mechanisms on the reservoir network, that is, node-attack and edge-attack, while in biological neural networks, the two types of attack may represent the apoptosis of neurons and the fracture of synapses respectively. In physical implementations, the two types correspond to failure of a single memristor and disconnection of the circuit between memristors respectively. It is shown that RC may lose efficacy under both attack mechanisms [Figure 2c,d].

**Mechanism** **1** (**Node-attack**)**.** *For a well-trained and autonomous RC, node-attack denotes the removal of certain node s and its all adjacent edges, which is performed by*

$$r_{k}:=0,\;\;\;W_{\mathrm{res}}^{:,s}:=0,\;\;\;W_{\mathrm{res}}^{s,:}:=0,\;\;\;W_{\mathrm{in}}^{:,s}:=0,\;\;\;W_{\mathrm{out}}^{s,:}:=0,$$

*where* $k>k^{*}$, $k^{*}$ *is the attack time, and the superscript “:” denotes the corresponding row/column.*

**Mechanism** **2** (**Edge-attack**)**.** *For a well-trained and autonomous RC, edge-attack denotes the removal of certain link from node i to node j, which is performed by*

$$W_{\mathrm{res}}^{ij}:=0.$$

In practice, the attack can be launched to a proportion of the nodes and/or edges according to certain rule.

### 2.3. Rc-Revive Strategies

We denote $W_{\mathrm{res}}$ and ${\tilde{W}}_{\mathrm{res}}$ as the reservoir matrix before and after attack respectively. To realize recovery in an energy-efficient manner, we revive RC’s performance by adjusting only a small fraction of the weights in ${\tilde{W}}_{\mathrm{res}}$, which are treated as values to be optimized to achieve minimal MSE, leading to the revived reservoir matrix $W_{\mathrm{res}}^{*}$.

Different optimization methods can be utilized to achieve the goal. In this study, Simulated Annealing (SA) [43] is used and integrated in our proposed reviving strategies to automatically find the optimal set of connections to impose adjustment, with the specific procedures presented in Algorithm 1.
**Algorithm 1 **Simulated Annealing-based recovery of RC**Input:** ${\tilde{W}}_{\mathrm{res}}$, E is a set of edges in ${\tilde{W}}_{\mathrm{res}}$ allowing adjusting depending on strategies, $k_{\max}$ is the maximum iterations, *N* is the number of edges perturbed each time during SA **Output:**
$W_{\mathrm{res}}^{*}$     MSE = MSE(${\tilde{W}}_{\mathrm{res}}$) 
    **for** 
$t=1\to k_{\max}$
 **do**

        Perturb *N* edges randomly from E by adding random(−1,1) to obtain $W_{\mathrm{res}}^{*}$
        ${\mathrm{MSE}}^{*}=\mathrm{MSE}\left(W_{\mathrm{res}}^{*}\right)$
        **if** ${\mathrm{MSE}}^{*}<\mathrm{MSE}$ **then**
            P=1
        **else**
            $P=\exp(\frac{-({\mathrm{MSE}}^{*}-\mathrm{MSE})}{0.95^{t}})$
        **end if**
        **if** P⩾random(0,1) **then**
            $\mathrm{MSE}={\mathrm{MSE}}^{*},{\tilde{W}}_{\mathrm{res}}=W_{\mathrm{res}}^{*}$
        **end if**
    **end for**

    $W_{\mathrm{res}}^{*}={\tilde{W}}_{\mathrm{res}}$


We first propose two strategies to revive RC from node-attack. Here the reservoir structure is preserved during the recovery process, that is, no new links is allowed to generate [Figure 1c]. As shown in the following Strategy 1 and 2, we choose the set of connections in a completely random manner or related to the attacked node respectively. In physical implementations, the selected set denotes partial connections between the memristors.

**Strategy** **1** (Full selection)**.** *The allowing set of edges here is defined as*

$$E_{F}:=\left\{\left(i,j\right)|i,j\neq s,{\tilde{W}}_{\mathrm{res}}^{ij}\neq 0\right\}.$$

**Strategy** **2** (Relevant selection)**.** *The allowing set of edges here is defined as*

$$\left.\begin{array}{c} E_{R}:=\left\{\left(i,j\right)|i,j\neq s,{\tilde{W}}_{\mathrm{res}}^{ij}\neq 0,W_{\mathrm{res}}^{is},W_{\mathrm{res}}^{si},W_{\mathrm{res}}^{js},W_{\mathrm{res}}^{sj}\;\mathrm{are}\;\mathrm{not}\;\mathrm{all}\;\mathrm{zero}\right\}. \end{array}\right.$$

In this study, we consider both fully and sparsely connected reservoir structures, while Strategy 1 and 2 become the same for the former case and we compare the two strategies for the latter case. Biologically, the relevant selection strategy is more ubiquitous since compensation always occurs at the neighbouring neurons [10,11,12].

We next consider the recovery of edge-attack. The above two strategies can be utilized in an analogous manner and achieve good recovery results. Here we propose another strategy allowing adding new links, which enriches the structure of the reservoir and represents the birth of new synapses. This strategy is more natural in physiology, but difficult to implement in digital devices.

**Strategy** **3** (Incremental selection)**.** *The allowing set of edges here contains all rest of the edges:*

$$E_{I}:=\left\{\left(i,j\right)|\left(i,j\right)\;\mathrm{is}\;\mathrm{not}\;\mathrm{attacked}\right\}.$$

*We trade off the selected edges by* γ *percentage of existing edges and* 1−γ *percentage of adding edges during SA.*

Note that retraining the RC may be considered as a solution. However, considering practical scenarios, retraining usually requires re-collecting a large amount of training data. Moreover, the operators of the devices often are not authorized to touch the system underpinnings [44], which imposes a requirement of adaptive recovery mechanism that does not need external interventions.

## 3. Result

We test and analyze the proposed methods in reservoir computers with m=100 reservoir neurons and leaky factor α=0.25, which is trained with the normalized *x* component of the benchmark Lorenz system:

(5) $$\left\{\begin{array}{c} \dot{x}=-\sigma x+\sigma y,\\ \dot{y}=rx-y-xz,\\ \dot{z}=-bz+xy \end{array}\right.$$

with σ=10,r=28,b=8/3 [45]. 1000 points with discretization time step 0.03 are used as training set, and the leading 100 reservoir states are discarded to eliminate the transient behavior. The input matrix $W_{\mathrm{in}}$ and the reservoir matrix $W_{\mathrm{res}}$ are generated with the elements randomly selected from [−1,1]. $W_{\mathrm{res}}$ is rescaled to a spectral radius of 0.9. We set the initial value of the SA procedure to be the reservoir state after attack, that is, ${\tilde{W}}_{\mathrm{res}}$, and update the temperature every 100 iterations with $k_{\max}=20,000$ maximum iterations.

### 3.1. Node-Attack and Recovery

Before attack, the RC is well-trained to a high prediction accuracy, see Figure 2a,b, while it loses efficacy when one randomly selected node is attacked [Figure 2c,d]. Strategy 1 is utilized with N=50 to fully connected RC and achieves good recovery result that significantly reducing the prediction errors [Figure 2e,f]. Notice that for fully connected RC, performing Strategy 2 is completely equivalent.

**Figure 3:**
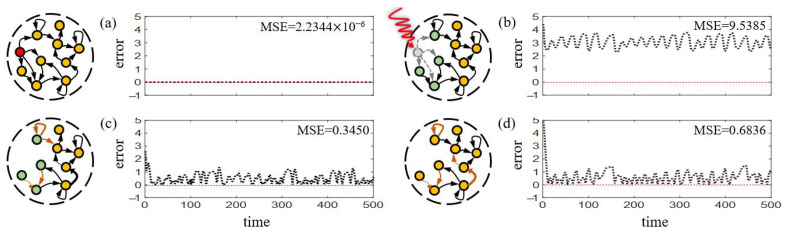
**Node-attack and recovery for sparsely connected RC.** The prediction errors for stages of (**a**) before attack, (**b**) attacked, (**c**) recovery with relevant selection, (**d**) recovery with full selection, respectively. Green nodes denote the neighbours of the attacked node. Here the MSE value denotes an average of 50 realizations.

For sparsely connected RC (here we allow 10% connections), both strategies are applied which successfully revive the performance to satisfactory extent [Figure 3]. However, Strategy 1 obtains relatively higher MSE (0.6836, Figure 3d) than the relevant selection strategy (0.3450, Figure 3c). Possible explanation lies in that adjacent links can be regarded as belonging to the same subgraph containing the attacked node, thus share similar local information, facilitating better recovery by adjusting them [46].

**Figure 4:**
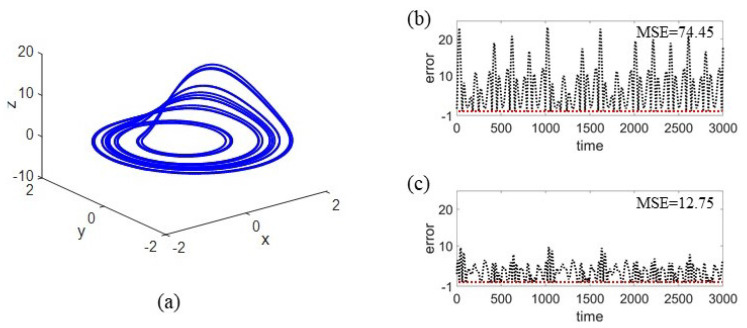
**Node-attack and recovery for fully connected RC in the task of reconstructing Rössler system.** (**a**) The original Rössler attractor. The reconstructing errors for stages of (**b**) attacked and (**c**) recovery are shown. Here the MSE value denotes an average of 50 realizations.

To demonstrate the mechanisms can be applied to various tasks, we additionally consider a system reconstructing task of the Rössler system:

(6) $$\left\{\begin{array}{c} \dot{x}=-\omega y-z,\\ \dot{y}=\omega x+\alpha y,\\ \dot{z}=\beta +z\left(x-\gamma \right) \end{array}\right.$$

with ω=1,α=0.2,β=0.4, and γ=5.7. Here normalized time series $x_{t}$ and $y_{t}$ are used to reconstruct the dynamics of $z_{t}$ and the parameter settings of RC are the same with the above experiment. We impose a node-attack to a fully connected RC and use Strategy 1 for recovery with N=50, and the experimental results are shown in Figure 4. Notice that after the attack, RC’s reconstructing ability is significantly weakened, with large MSE of 74.45. After taking the strategy, the reconstructing successfully recovers with the MSE decreasing to 12.75.

### 3.2. Edge-Attack and Recovery

**Figure 5:**
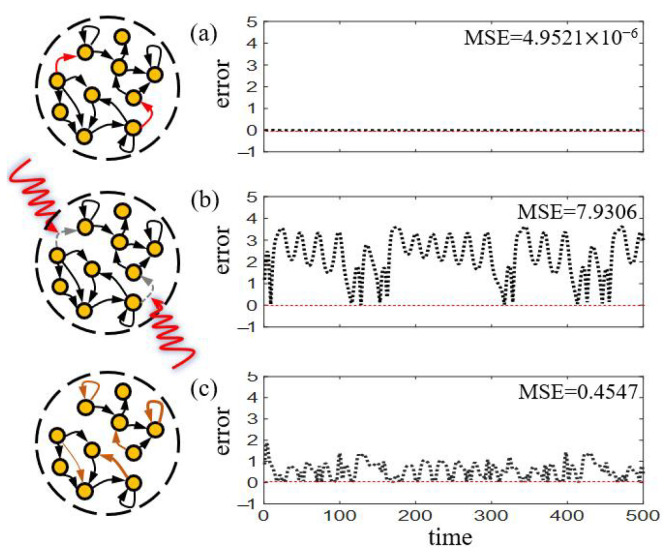
**Edge-attack and recovery for fully connected RC.** The prediction errors for stages of (**a**) before attack, (**b**) attacked, (**c**) recovery, respectively. Here two edges are attacked for illustration. Here the MSE value denotes an average of 50 realizations.

**Figure 6:**
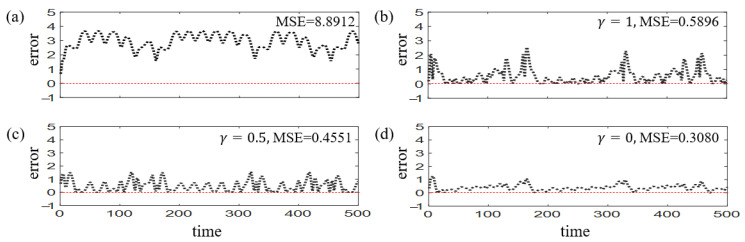
**Edge-attack and recovery for sparsely connected RC.** The prediction errors for stages of (**a**) attacked, (**b**) recovery with Strategy Section 2.3 (γ=1), (**c**) recovery with Strategy Section 2.3 (γ=0.5), (**d**) recovery with Strategy Section 2.3 (γ=0), respectively. Here the MSE value denotes an average of 50 realizations.

Here attack is launched to 10% randomly selected edges. We apply Strategy 3 with γ=1 and N=50 to a fully connected RC and present the results in Figure 5a–c, which also reduces the prediction errors significantly and revives the RC to a healthy condition. Notice that for fully connected RC, γ<1 leads to the rebirth of the attacked edges, which is not allowed in our settings. While for sparsely connected RC, we analyze the recovery effects with different choices of γ. Notice that γ=0 denotes the circumstance that recovery is reached only by generating new edges and preserving all the existing ones, which produces the MSE of 0.3080 [Figure 6b]. When γ increases to 0.5, denoting a mixing strategy of generating new edges and adjusting existing edges, recovery MSE also increases to 0.4551 [Figure 6c]. Additionally, the strategy with γ=1 produces the highest MSE of 0.5896 [Figure 6d]. The above MSE values are obtained through averaging the results of 50 realizations. The experiments show that in a sparsely connected RC, adding new edges becomes the best recovery strategy, which represents an enrichment of the reservoir’s structure. This is in accordance with biological nervous system, in which compensation is always reached by generating new synapses [10,11,12].

## 4. Discussion

### 4.1. Ineffectiveness of Precaution to Reservoir

To avoid performance collapsing under attack, precautionary actions may be taken. For example, if we can identify the most essential nodes/edges in advance, protections can be imposed to these targets. Usually, there are many methods/measurements from complex network theory to evaluate the importance of nodes based on their local or global information, e.g., Degree Centrality [47], Node Strength [48], Betweenness Centrality [49], PageRank [50], and so forth. For a fully connected reservoir, all nodes have indistinguishable evaluations, preventing the emergence of the key nodes. Here we show in the framework of sparse reservoir computer, the performance loss under node-attack is also irrelevant with node’s importance measurements. We test the linear relationship between MSE under node-attack and several measurements of the node, and list the results in Table 1, demonstrating all nodes are of similar importance in the reservoir. Therefore, taking precautionary actions is difficult, and recovery strategies proposed in this work are of great significance. In addition, previous experiments show that although the reservoir structure is initially randomly chosen, the performance is quite sensitive to small perturbations in the network structure, further demonstrating the significance of this work.

### 4.2. Echo State Property vs. Attack and Recovery

**Figure 7:**
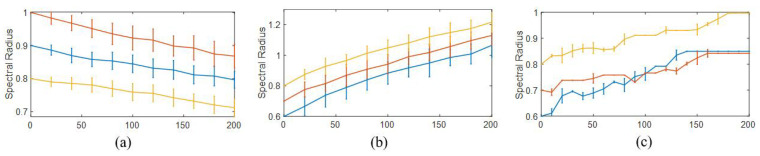
**Variation of spectral radius during attack and recovery.** (**a**) A decreasing trend from differently selected initial values of the spectral radius during edge-attack, harming the performance of RC. (**b**) The spectral radius gradually increases as the network gets denser, but will exceed 1 eventually, violating the ESP and failing the performance. (**c**) With our framework, the spectral radius stabilizes at around 1 and the network stops growing automatically, guaranteeing the best performance of recovery. The horizontal axis show the numbers of added/removed edges. Error bar denotes standard deviation of 20 realizations.

The spectral radius of the reservoir matrix plays crucial role in determining the performance of RC by ensuring the Echo State Property and balancing memory capacity and nonlinearity [51]. We are interested in the variation of the spectral radius, thus the ESP, during attack and recovery. A rigorous theory characterizing this variation lies in the spectral graph theory and can be referred to reference [52]. Here we perform numerical analysis using 100×100 randomly and sparsely connected reservoir network matrix rescaled to different initial spectral radius and randomly remove an increasing proportion edges. As shown in Figure 7a, the spectral radius has a decreasing trend following the removing procedure, harming the performance of RC, as demonstrated in [24] that RC runs most effectively with a spectral radius close to 1. However the ESP remains satisfied.

For the recovery, we successively added new edges to the reservoir network, and present the results in Figure 7b. We find the spectral radius gradually increases as the network gets denser, but will exceed 1 eventually, violating the ESP and failing the performance. However, in our framework, utilizing Strategy 3 together with the Simulated Annealing optimization produces Figure 7c, which shows that the spectral radius stabilizes at around 1 and the network stops growing automatically, guaranteeing the best performance of recovery.

**Figure 8:**
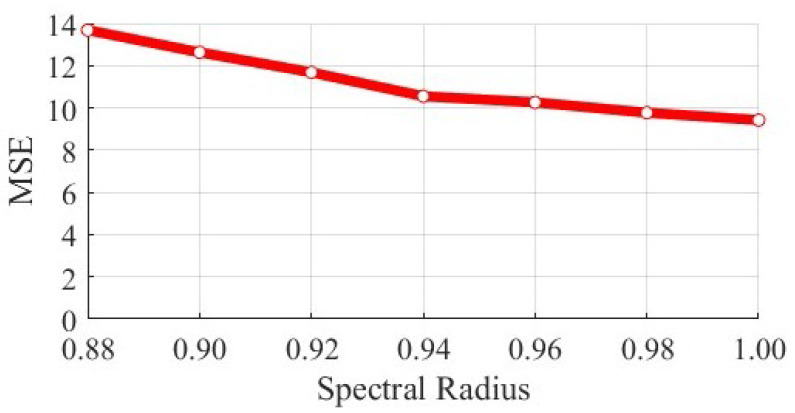
**Variation of MSE during spectral radius adjustment.** MSE gradually decreases with the increase in the spectral radius, but the recovery is inefficient and the MSE after recovery remains large compared to our proposed strategies.

Meanwhile, we experimentally verify that whether the recovery can be achieved by adjusting the spectral radius. We rescale the fully connected reservoir matrix being node-attack, and find that the MSE shows a slow decreasing trend with the turning up of the spectral radius (still remains large after adjustment, see Figure 8). Nevertheless, adjusting the spectral radius requires an overall manipulation of the whole matrix, which is time-consuming compared to our proposed strategies.

### 4.3. How to Choose Recovery Strategies

**Figure 9:**
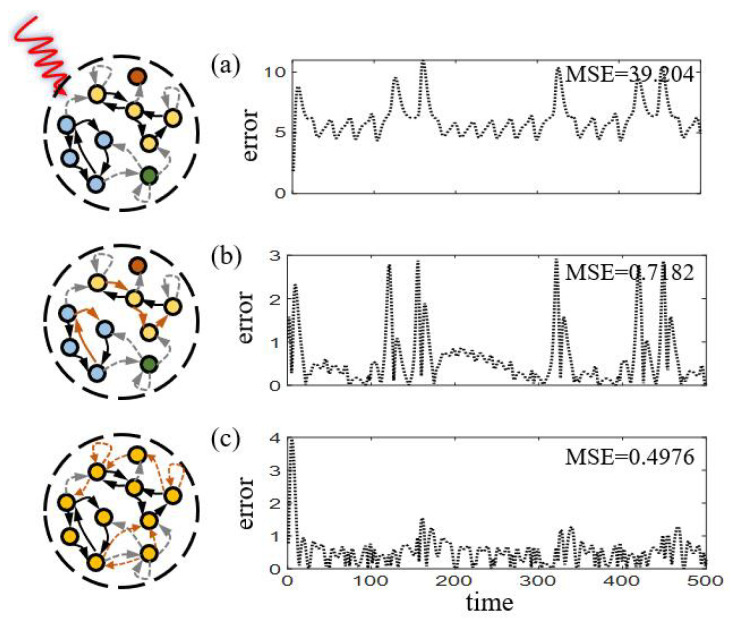
**Different choices of recovery strategies according to the reservoir connectedness.** The prediction errors for stages of (**a**) attacked, (**b**) recovery with Strategy 2, (**c**) recovery with Strategy 3 (γ=0), respectively. Here different colors of the nodes denote their connectedness and the MSE value denotes an average of 50 realizations.

Exposing to different attacked scenarios, we analyze the proper selection of recovery strategies. Two criteria, reservoir connectedness [53] and attacked edge betweenness centrality [54], are adopted to determine the optimal strategy. Here we compare two attacked scenarios for a sparsely connected RC, with its healthy state presented in Figure 2a. The first case (attack 90% connections) renders the reservoir separating into several connected components, significantly harming its prediction ability [Figure 9a]. Strategy 2 is applied with *s* adjusting to be the vertexes of the attacked edges, decreasing the MSE to 0.7182 [Figure 9b]. As comparison, Strategy 3 with γ=0 is applied and achieves generally better recovery results. At optimal case that the reservoir returns to be connected, smaller MSE of 0.4976 is achieved [Figure 9c]. However, for the second case of attack that does not harm the reservoir’s connectedness, the two strategies have similar results, with MSE values of 0.8979 and 0.8176 respectively.

These results demonstrate the importance of the reservoir connectedness in selecting optimal strategies. Generally, against massive attacks, adding new edges to restore the reservoir connectedness can be preferentially chosen, as connectedness plays crucial role in ensuring the network’s proper functioning [53].

**Figure 10:**
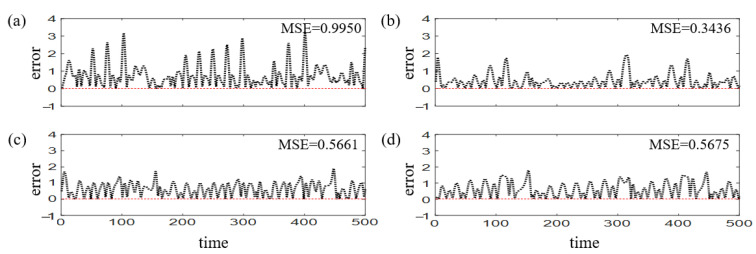
**Different choices of recovery strategies according to the attacked edge betweenness centrality.** When the betweenness of the attacked edges is high, the prediction errors for stages of (**a**) recovery with Strategy 2, (**b**) recovery with Strategy 3. When the betweenness of the attacked edges is low, the prediction errors for stages of (**c**) recovery with Strategy 2, (**d**) recovery with Strategy 3. Here the MSE value denotes an average of 50 realizations.

With the same configurations, we compare Strategy 2 and Strategy 3 respectively in two attacked scenarios with the average betweenness of the attacked edges greater (3.8119) or smaller (3.0427) than the average betweenness of the reservoir before attack (3.414). Results in Figure 10 show the preference of Strategy 3 when the attacked edges are relatively important and indiscriminate selection of Strategy 2 and 3 for less important attacked edges. In fact, a higher value of the betweenness implies the criticality of the edges [55], and adding new edges avails recovery when crucial edges are attacked.

### 4.4. Minor Adjusting Is Sufficient for Recovery

**Figure 11:**
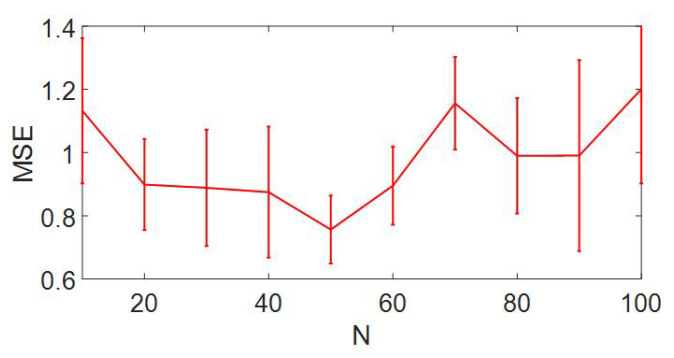
**Efficacy of recovery v.s. number of adjusted edges for fully connected RC under node-attack.** Lowest MSE is reached at N=50, demonstrating that minor adjusting is sufficient for recovery. Error bar denotes standard deviation of 20 realizations.

In practice, efficient recovery is expected to achieve at minimized cost, which in our study is reflected in the number of edges perturbed (*N*) during optimization, yielding a trade-off between performance and cost. Here we search for an optimized *N* for a fully connected RC under node-attack with full selection recovery strategy. The experimental configurations are the same with Figure 2, but varying *N* from 10 to 100. As compared to the network size (100×100), recovery can be reached with extremely minor adjusting and low cost. As shown in Figure 11, lowest MSE is reached at N=50 and increasing the number of adjusted edges does not benefit the efficacy of recovery. This result further demonstrates the broad applicability and high efficiency of our proposed framework.

### 4.5. Attack and Recovery in Other Neural Networks

Reservoir computing, as a specific variant of RNN, is subject to not only attacks on network structure but also adversarial attacks, while the latter is more commonly considered in traditional NN, e.g., Fast Gradient Sign Method (FGSM) deteriorates the performance in tasks of time series prediction and graph node classification [56,57], DeepFake causes the face recognition model to misclassify [58,59,60], and so forth. Adversarial attacks on RC also represent a promising topic which will be included in our future work, including on other variants of RNN, such as LSTM [61] and GRU [62]. Moreover, attacks on network structure of Deep Neural Networks (DNN) are regarded as training tricks, instead of attacks, to improve the performance due to the redundant structures in DNN.

## 5. Conclusions

In this paper, inspired by the biological neural compensation mechanism in brains, we proposed a framework of reviving attacked reservoir computers, consisting of several strategies directed to different types of attacks. All the strategies achieved sound recovery results. The analysis further brings inspiring insights, that:

(1) Adjusting adjacent neurons/synapses is more effective than distant ones;
(2) Enriching the reservoir network is more effective than adjusting existing edges;
(3) Reservoir connectedness and attacked edge betweenness centrality are crucial criteria in choosing optimal recovery strategies; and
(4) Minor adjustments are sufficient for recovery.

Future work includes incorporating advanced optimization algorithms, theoretical analysis on the choice of adjusting connections, and designing more adaptive strategies. The proposed attack and recovery strategies can be generalized to more variants of RNN, including LSTM and GRU. This work provided a practical framework to improve the robustness of reservoir computers, and a vehicle towards broader types of neural networks.

## Figures and Tables

**Table 1:** Relationship between node-attack MSE and importance measurements (y=ax+b).

Importance Measurement	a	$R^{2}$-Score
Degree Centrality	−0.32±0.65	0.03
Node Strength	−0.51±0.72	0.06
Betweenness Centrality	+0.28±0.52	0.12
PageRank	−0.30±0.81	0.07

## Data Availability

Data sharing is not applicable to this article as no new data were created or analyzed in this study.
